# Feasibility of veno-arterial extracorporeal life support in awake patients with cardiogenic shock

**DOI:** 10.1093/icvts/ivae148

**Published:** 2024-08-20

**Authors:** Iris Feng, Sameer Singh, Serge S Kobsa, Yanling Zhao, Paul A Kurlansky, Ashley Zhang, Anna J Vaynrub, Justin A Fried, Koji Takeda

**Affiliations:** Division of Cardiothoracic and Vascular Surgery, Department of Surgery, Columbia University Irving Medical Center, New York, New York, USA; Division of Cardiothoracic and Vascular Surgery, Department of Surgery, Columbia University Irving Medical Center, New York, New York, USA; Division of Cardiac Surgery, Department of Surgery, Keck School of Medicine at University of Southern California, Los Angeles, CA, USA; Division of Cardiothoracic and Vascular Surgery, Department of Surgery, Columbia University Irving Medical Center, New York, New York, USA; Division of Cardiothoracic and Vascular Surgery, Department of Surgery, Columbia University Irving Medical Center, New York, New York, USA; Department of Surgery, Center of Innovation and Outcomes Research, Columbia University, New York, NY, USA; Division of Cardiothoracic and Vascular Surgery, Department of Surgery, Columbia University Irving Medical Center, New York, New York, USA; Division of Cardiothoracic and Vascular Surgery, Department of Surgery, Columbia University Irving Medical Center, New York, New York, USA; Division of Cardiology, Department of Medicine, Columbia University Irving Medical Center, New York, NY, USA; Division of Cardiothoracic and Vascular Surgery, Department of Surgery, Columbia University Irving Medical Center, New York, New York, USA

**Keywords:** Awake extracorporeal membrane oxygenation, Mechanical ventilation, Extracorporeal membrane oxygenation, Cardiogenic shock, Mechanical circulatory support, Heart failure

## Abstract

**OBJECTIVES:**

This study sought to demonstrate outcomes of veno-arterial extracorporeal life support (VA-ECLS) in non-intubated (‘awake’) patients with cardiogenic shock, as very few studies have investigated safety and feasibility in this population.

**METHODS:**

This was a retrospective review of 394 consecutive VA-ECLS patients at our institution from 2017 to 2021. We excluded patients cannulated for indications definitively associated with intubation. Patients were stratified by intubation status at time of cannulation and baseline differences were balanced by inverse probability of treatment weighting. The primary outcome was in-hospital mortality while secondary outcomes included adverse events during ECLS and destination at discharge.

**RESULTS:**

Out of 135 patients in the final cohort, 79 were intubated and 56 were awake at time of cannulation. All awake patients underwent percutaneous femoral cannulation with technical success of 100% without intubation. Indications for VA-ECLS in awake patients included acute decompensated heart failure (64.3%), pulmonary hypertension or massive pulmonary embolism (12.5%), myocarditis (8.9%) and acute myocardial infarction (5.4%). After adjustment, awake and intubated patients had similar ECLS duration (7 vs 6 days, *P* = 0.19), in-hospital mortality (39.6% vs 51.7%, *P* = 0.28), and rates of various adverse events. Intubation status was not a significant risk factor for 90-day mortality (hazard ratio [95% confidence interval]: 1.26 [0.64, 2.45], *P* = 0.51) in multivariable analysis. Heart transplantation (15.1% vs 4.9%) and ventricular assist device (17.4% vs 2.2%) were more common destinations at discharge in awake patients than intubated patients (*P* = 0.02).

**CONCLUSIONS:**

Awake VA-ECLS is safe and feasible with comparable outcomes as intubated counterparts in select cardiogenic shock patients.

## Graphical abstract

**Figure:**
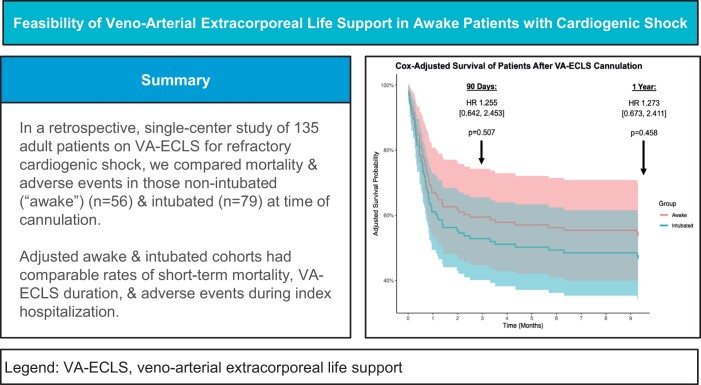

## INTRODUCTION

Veno-arterial extracorporeal life support (VA-ECLS) has increasingly become an important tool in the treatment of patients with refractory cardiogenic shock (RCS) [1]. By rapidly providing biventricular circulatory support, it can effectively be used as a bridge to recovery or advanced heart failure therapies such as durable ventricular assist device (VAD) or transplantation [2, 3]. However, outcomes for patients with RCS and in particular those requiring VA-ECLS remain poor with high rates of device-associated complications as well as significant morbidity and mortality [4].

Traditionally, patients requiring VA-ECLS are sedated and mechanically ventilated due to concomitant respiratory failure in the setting of RCS and for undergoing the cannulation procedure itself. Additionally, concern for cannula dislodgement often results in higher levels of sedation and immobilization of patients. Unfortunately, this may compromise patient care and lead to further complications. Prolonged mechanical ventilation, though sometimes necessary, can lead to barotrauma, pneumothorax and ventilator-associated pneumonia [5, 6]. Additionally, positive-pressure ventilation can have a negative effect on cardiac output. General anaesthetic sedation and the intubation process can also lead to a higher degree of vasopressor support and haemodynamic collapse. Finally, prolonged immobilization in these patients can lead to debilitation and a decreased likelihood of successful bridging to advanced therapies [7].

Recently, the use of veno-venous ECLS in patients with end-stage lung disease as a first-line therapy or alternative to mechanical ventilation has gained popularity as a means of bridging lung transplantation [8, 9]. Implementation of VA-ECLS in non-ventilated patients, commonly referred to as ‘awake ECLS’ in the literature, may also have particular advantages in patients with RCS. Besides avoidance of ventilator-associated complications, awake VA-ECLS also allows real-time neurological monitoring in these critically ill patients that may facilitate evaluation for subsequent therapies, such as VAD or heart transplantation [10]. Unfortunately, the use of VA-ECLS in awake adult patients remains under-investigated with the majority of data limited to small case series [11–15]. Therefore, the purpose of this study was to examine outcomes of awake patients that required VA-ECLS support for RCS at a single, high-volume ECLS center.

## PATIENTS AND METHODS

### Ethical statement

This study was approved by the Columbia University Institutional Review Board (Protocol AAAU2877, approved on 21 December 2023) with waiver of consent due to existing hospital records.

### Patient cohorts

A retrospective review of the electronic medical record was performed for 394 consecutive patients requiring VA-ECLS for RCS between 2017 and 2021 at our institution. During this study period, we performed ‘awake’ ECLS for all patients who were not intubated before cannulation. These patients underwent percutaneous femoral cannulation at the bedside under local anaesthesia with technical success of 100%.

Exclusion criteria were patients cannulated for extracorporeal cardiopulmonary resuscitation (*n* = 90), post-cardiotomy shock (*n* = 126), or primary graft failure after heart transplant (*n* = 43) as these patients definitively required intubation and sedation. Patients were then divided into intubated (*n* = 79) and non-intubated (‘awake’) (*n* = 56) groups based on intubation status at the time of cannulation. None of the patients in the intubated cohort were electively intubated exclusively for the cannulation procedure. Sub-analyses, where specified, further stratified awake and intubated groups into four cohorts based on whether or not intubation status changed during the VA-ECLS course: (i) remained awake during entire VA-ECLS course, (ii) awake during cannulation then underwent emergent intubation while on VA-ECLS, (iii) remained intubated during entire VA-ECLS course and (iv) intubated during cannulation then extubated before decannulation.

Data for each patient was collected from the electronic medical record, including basic demographics, co-morbidities, baseline laboratory values, aetiology of cardiogenic shock, Society for Cardiovascular Angiography and Interventions (SCAI) shock classification at the time of VA-ECLS initiation and presence of intra-aortic balloon pump or Impella device. Etiologies of cardiogenic shock included acute myocardial infarction, acute decompensated heart failure, massive pulmonary embolism or pulmonary hypertensive crisis, ventricular arrythmia and myocarditis. Serum creatinine level measured immediately prior to VA-ECLS cannulation was utilized in the 2021 Chronic Kidney Disease Epidemiology Collaboration equation below to calculate baseline estimated glomerular filtration rate [16]. The equation utilizes 3 variables: gender, age and serum creatinine.

$${\text{eGFR}}_{\text{Cr}}=142\times \text{min}{\left(S_{\text{Cr}}/\kappa ,1\right)}^{\alpha }\times \text{max}{\left(S_{\text{Cr}}/\kappa ,\;1\right)}^{-1200}\times 0.9938^{\text{Age}}\times 1.012\;\left[\text{if female}\right]$$

where S_Cr_ is serum creatinine in mg/dl, κ is 0.7 for females and 0.9 for males, α is −0.241 for females and −0.302 for males, min indicates the minimum of S_Cr_/κ or 1 and max indicates the maximum of S_Cr_/κ or 1.

Clinical characteristics and outcomes were extracted from retrospective review of the electronic medical record and compared between groups. Missing data in baseline variables are listed in Supplementary Material, Table S1. These variables were not included in modelling analyses due to substantial missingness. The date of and status at most recent follow-up were collected based on the most recent encounter available in the medical record. The median follow-up time was 101 days, with interquartile range (IQR) of 18– to 957 days. The primary outcome of this study was all-cause mortality.

### Statistical analysis

R software was used for all statistical analyses and illustrations, with details of statistical packages listed in Supplementary Material, Table S2. Continuous variables were assessed for normality by the Shapiro–Wilk test and reported as median [interquartile range] as all were non-normally distributed. Categoric variables are presented as proportions with absolute numbers. Differences between groups were measured using the Chi Square or Fisher’s exact test for categoric variables and the Mann–Whitney *U*-test for continuous variables. Kaplan–Meier curves were created for unadjusted 4-group comparisons of all-cause mortality, with *P*-values generated by log-rank test.

To adjust for baseline differences between awake and intubated groups, we performed inverse probability of treatment weighting (IPTW) with a covariate-balancing propensity score method. A binary logistic regression based on age, body mass index, intra-aortic balloon pump, and aetiology of cardiogenic shock was used to generate a propensity score for each patient. Trimming was applied at the 2^nd^ and 98^th^ percentiles of the propensity score distribution (Supplementary Material, Fig. S1) where the lowest weight was 0.452 and the highest weight was 13.362. A standardized mean difference (SMD) < 0.2 was considered an indicator of adequate balance [17], with distribution of SMD values before and after IPTW displayed in Supplementary Material, Fig. S2. Multivariable Cox regression modelling of IPTW-adjusted awake and intubated cohorts was utilized to assess the effect of intubation status at the time of cannulation on all-cause mortality while further adjusting for the effect of diabetes and SCAI classification. Death events occurred in 58 and 65 patients at 90 days and 1 year post-cannulation, respectively. Cox-adjusted curves were also generated to display 90-day and 1-year survival of awake versus intubated cohorts. Logistic regression modelling of adjusted cohorts was utilized to assess whether intubation status at the time of cannulation was a significant risk factor for developing pneumonia (58 events) or requiring tracheostomy (32 events) while on VA-ECLS or after decannulation before discharge, while further adjusting for effect of age, chronic obstructive pulmonary disease and smoking history. The proportional hazard assumption was evaluated by the Schoenfeld residuals plot and found not to be violated. No collinearity was found among variables in the multivariable models given all variance inflation factors < 2. Results are presented as hazard ratio or odds ratio with a corresponding 95% confidence interval (CI) calculated using robust standard errors to account for clustering within matched sets.

## RESULTS

A total of 135 patients were included in this study. Of these, 79 were intubated (58.5%) and 56 were awake (41.5%) at time of VA-ECLS cannulation. In unadjusted patient cohorts, awake patients were younger (54.5 vs 61.0 years, SMD = 0.432) and had a higher frequency of SCAI stage D (76.8% vs 36.7%, SMD = 0.957) and intra-aortic balloon pump (58.9% vs 29.1%, SMD = 0.379) compared to intubated patients. Awake patients also had lower body mass index (26.9 vs 30.4, SMD = 0.521) and lactate (2.1 vs 3.0, SMD = 0.406) at baseline. Indications for VA-ECLS in awake versus intubated patients included acute decompensated heart failure (64.3% vs 32.9%), pulmonary hypertensive crisis or massive pulmonary embolism (12.5% vs 11.4%), acute myocardial infarction (5.4% vs 32.9%), myocarditis (8.9% vs 2.5%) and ventricular arrhythmia (1.8% vs 10.1%) (SMD = 0.999) (Table 1).

**Table 1:** Baseline characteristics of intubated and awake cohorts, before and after inverse probability of treatment weighting (IPTW) adjusting for age, BMI, IABP and aetiology of cardiogenic shock (Entry in bold)

	Unadjusted			After IPTW		
Variable	Awake (*n* = 56)	Intubated (*n* = 79)	SMD	Awake (*n* = 52.1)	Intubated (*n* = 77.6)	SMD
**Age**	**54.5 [45.3, 64.0]**	**61.0 [53.0, 67.0]**	**0.432**	**57.6 [47.0, 64.6]**	**57.9 [48.0, 65.7]**	**0.079**
Male gender	37 (66.1)	54 (68.4)	0.049	32.9 (63.2)	49.9 (64.3)	0.023
**BMI (kg/m^2^)**	**26.9 [22.3, 30.4]**	**30.4 [27.1, 35.4]**	**0.521**	**28.1 [22.3, 34.1]**	**29.1 [26.5, 33.6]**	**0.043**
BSA (m^2^)	1.9 [1.8, 2.1]	2.0 [1.9, 2.2]	0.359	2.0 [1.8, 2.2]	2.0 [1.9, 2.2]	0.087
Past medical history						
Coronary artery disease	24 (42.9)	46 (58.2)	0.311	23.5 (45.2)	45.8 (59.0)	0.280
Congestive heart failure	38 (67.9)	39 (49.4)	0.382	30.6 (58.7)	40.0 (51.6)	0.144
Hyperlipidaemia	31 (55.4)	54 (68.4)	0.270	30.9 (59.4)	48.3 (62.2)	0.058
Diabetes	21 (37.5)	31 (39.2)	0.036	25.8 (49.5)	23.3 (30.0)	0.407
Hypertension	43 (76.8)	61 (77.2)	0.010	40.5 (77.8)	54.0 (69.5)	0.188
COPD	3 (5.4)	7 (8.9)	0.137	2.2 (4.2)	6.8 (8.8)	0.189
Chronic kidney disease	25 (44.6)	27 (34.2)	0.215	19.1 (36.6)	25.7 (33.2)	0.073
Smoking	26 (46.4)	37 (46.8)	0.008	29.3 (56.2)	35.9 (46.2)	0.202
CVA	9 (16.1)	10 (12.7)	0.097	6.9 (13.2)	9.4 (12.1)	0.034
**Aetiology of shock**			**0.999**			**0.199**
** Acute MI**	**3 (5.4)**	**26 (32.9)**		**9.0 (17.2)**	**17.0 (21.9)**	
** ADHF**	**36 (64.3)**	**26 (32.9)**		**24.9 (47.9)**	**34.2 (44.1)**	
** Pulmonary**	**7 (12.5)**	**9 (11.4)**		**8.6 (16.6)**	**9.7 (12.5)**	
** Ventricular arrythmia**	**1 (1.8)**	**8 (10.1)**		**2.1 (4.1)**	**5.2 (6.8)**	
** Myocarditis**	**5 (8.9)**	**2 (2.5)**		**3.2 (6.1)**	**5.0 (6.4)**	
** Other**	**4 (7.1)**	**8 (10.1)**		**4.2 (8.1)**	**6.5 (8.3)**	
Impella	33 (58.9)	23 (29.1)	0.630	14.3 (27.4)	18.5 (23.9)	0.081
**IABP**	**10 (17.9)**	**27 (34.2)**	**0.379**	**21.4 (41.0)**	**31.8 (41.0)**	**0.001**
SCAI stage			0.957			0.933
C	9 (16.1)	20 (25.3)		7.6 (14.5)	17.5 (22.6)	
D	43 (76.8)	29 (36.7)		41.6 (80.0)	32.4 (41.8)	
E	4 (7.1)	30 (30.8)		2.9 (5.5)	27.7 (35.7)	
Echocardiography						
Moderate/severe MR	15 (57.7%)	14 (36.8%)	0.427	11.5 (45.4)	16.6 (42.9)	0.051
LVEF (%)	15.0 [14.0, 25.0]	20.0 [15.0, 30.0]	0.194	19.1 [14.3, 35.0]	20.0 [15.0, 36.8]	0.141
LVEDD (cm)	6.2 [4.8, 7.1]	5.6 [4.9, 6.3]	0.360	5.6 [4.4, 7.0]	5.5 [4.8, 6.5]	0.165
Haemodynamics						
MAP (mmHg)	72.0 [64.0, 78.0]	70.0 [65.5, 81.5]	0.095	71.5 [65.0, 79.3]	70.0 [64.0, 78.8]	0.014
CO (fick)	2.7 [2.2, 3.9]	4.0 [3.2, 4.6]	0.738	2.7 [2.1, 3.9]	4.1 [3.3, 4.6]	0.852
CI (fick)	1.5 [1.1, 1.9]	1.9 [1.5, 2.3]	0.691	1.5 [1.1, 1.9]	1.8 [1.5, 2.3]	0.833
CVP (mmHg)	17.5 [14.2, 21.0]	17.5 [14.0, 23.0]	0.020	17.0 [10.9, 21.0]	17.0 [13.0, 21.3]	0.044
MPAP (mmHg)	39.5 [29.8, 49.2]	32.0 [25.0, 40.0]	0.468	37.0 [28.0, 44.0]	33.0 [24.0, 40.9]	0.381
PCWP (mmHg)	27.0 [24.2, 35.5]	27.5 [23.5, 33.8]	0.111	27.0 [24.0, 36.1]	27.0 [23.6, 32.8]	0.158
PVR (woods units)	1.6 [1.3, 1.8]	1.8 [1.1, 2.2]	0.375	1.4 [0.5, 1.8]	1.7 [0.8, 2.2]	0.418
Labs						
P_a_CO_2_ (mmHg)	33.8 [28.3, 40.1]	38.0 [33.5, 42.2]	0.650	35.5 [30.0, 40.6]	38.7 [34.6, 46.1]	0.600
SvO_2_ (%)	45.6 [38.3, 57.8]	58.7 [46.0, 68.8]	0.844	47.4 [38.8, 58.7]	64.4 [45.8, 73.0]	0.926
P_a_O_2_/FiO_2_	306.4 [186.1, 389.4]	217.3 [106.0, 348.5]	0.476	273.8 [168.8, 378.0]	259.6 [106.8, 361.5]	0.193
Lactate (mmol/l)	2.1 [1.3, 4.1]	3.0 [1.7, 6.6]	0.406	2.2 [0.9, 4.5]	2.5 [1.6, 6.0]	0.353
Hematocrit (%)	34.5 [30.9, 39.2]	32.4 [27.0, 39.7]	0.013	33.7 [29.6, 39.1]	33.1 [27.6, 41.2]	0.003
eGFR (mL/min)	47.5 [32.9, 60.6]	39.4 [27.8, 63.0]	0.182	47.5 [34.3, 60.1]	39.2 [29.0, 75.0]	0.093

Categorical data are reported as value (%). Continuous data are reported as median [interquartile range].

ADHF: acute decompensated heart failure; BMI: body mass index; BSA: body surface area; CI: cardiac index; CO: cardiac output; CVA: cerebrovascular accident; CVP: central venous pressure; COPD: chronic obstructive pulmonary disease; eGFR: estimated glomerular filtration rate; ECLS: extracorporeal life support; IABP: intra-aortic balloon pump; LVEDD: left ventricular end-diastolic diameter; LVEF: left ventricular ejection fraction; MAP: mean arterial pressure; MI: myocardial infarction; MPAP: mean pulmonary arterial pressure; P_a_CO_2_: partial pressure of arterial carbon dioxide; PCWP: pulmonary capillary wedge pressure; PVR: pulmonary vascular resistance; SvO_2_: mixed venous oxygen saturation; P_a_O_2_/FiO_2_: partial pressure of arterial oxygen to fraction of inspired oxygen ratio; SCAI: Society for Cardiovascular Angiography and Interventions classification of cardiogenic shock; SMD: standardized mean difference.

After adjusting for several important baseline differences with IPTW (Table 1), awake and intubated patients had similar VA-ECLS duration (7 vs 6 days, *P* = 0.191), in-hospital mortality (39.6% vs 51.7%, *P* = 0.281), and frequency of various adverse events during hospitalization, although awake patients trended towards lower rates of tracheostomy during or after VA-ECLS (11.6% vs 25.2%, *P* = 0.05). During their VA-ECLS run, 14.8% of awake patients required emergent intubation while 20.4% of intubated patients were extubated before decannulation occurred (*P* < 0.001). Moreover, heart transplantation (15.1% vs 4.9%) and VAD (17.4% vs 2.2%) were more common destinations post-VA-ECLS among awake patients compared to those who were intubated (*P* = 0.016) (Table 2).

**Table 2:** Clinical outcomes of inverse probability treatment weighted intubated and awake patients

Outcome	Awake (*n* = 52.05)	Intubated (*n* = 77.62)	*P*-value
VA-ECLS duration (days)	7.0 [4.0, 11.0]	6.0 [3.0, 9.0]	0.191
Changed intubation status	7.7 (14.8)	15.8 (20.4)	<0.001*
Destination at discharge			0.016*
Death	20.6 (39.6)	40.2 (51.7)	
Recovery	14.5 (27.9)	31.9 (41.1)	
Transplant	7.9 (15.1)	3.8 (4.9)	
VAD	9.0 (17.4)	1.7 (2.2)	
In-hospital mortality	20.7 (39.6)	40.1 (51.7)	0.281
Required LV unloading	27.0 (51.8)	46.5 (59.8)	0.452
Adverse events			
Distal perfusion cannula	19.5 (37.6)	39.3 (50.6)	0.232
Limb ischaemia surgery	0.0 (0.0)	0.6 (0.8)	0.420
CVVH initiation	9.9 (19.1)	27.8 (36.3)	0.106
CVA	3.8 (7.3)	5.3 (6.8)	0.898
AKI	15.4 (29.6)	26.7 (34.4)	0.638
Bleeding	17.6 (33.8)	27.8 (35.8)	0.832
PNA during ECLS	9.3 (17.9)	17.0 (21.9)	0.664
PNA during or after ECLS	21.0 (40.4)	30.3 (39.0)	0.893
Trach during ECLS	1.7 (3.2)	4.4 (5.7)	0.550
Trach during or after ECLS	6.0 (11.6)	19.3 (25.2)	0.053

Categorical data are reported as value (%). Continuous data are reported as median [interquartile range].

* *P* < 0.05.

AKI: acute kidney injury; CVA: cerebrovascular accident; CVVH: continuous veno-venous hemofiltration; ECLS: extracorporeal life support; PNA: pneumonia; VAD: ventricular assist device; SMD: standardized mean difference.

**Figure 1:**
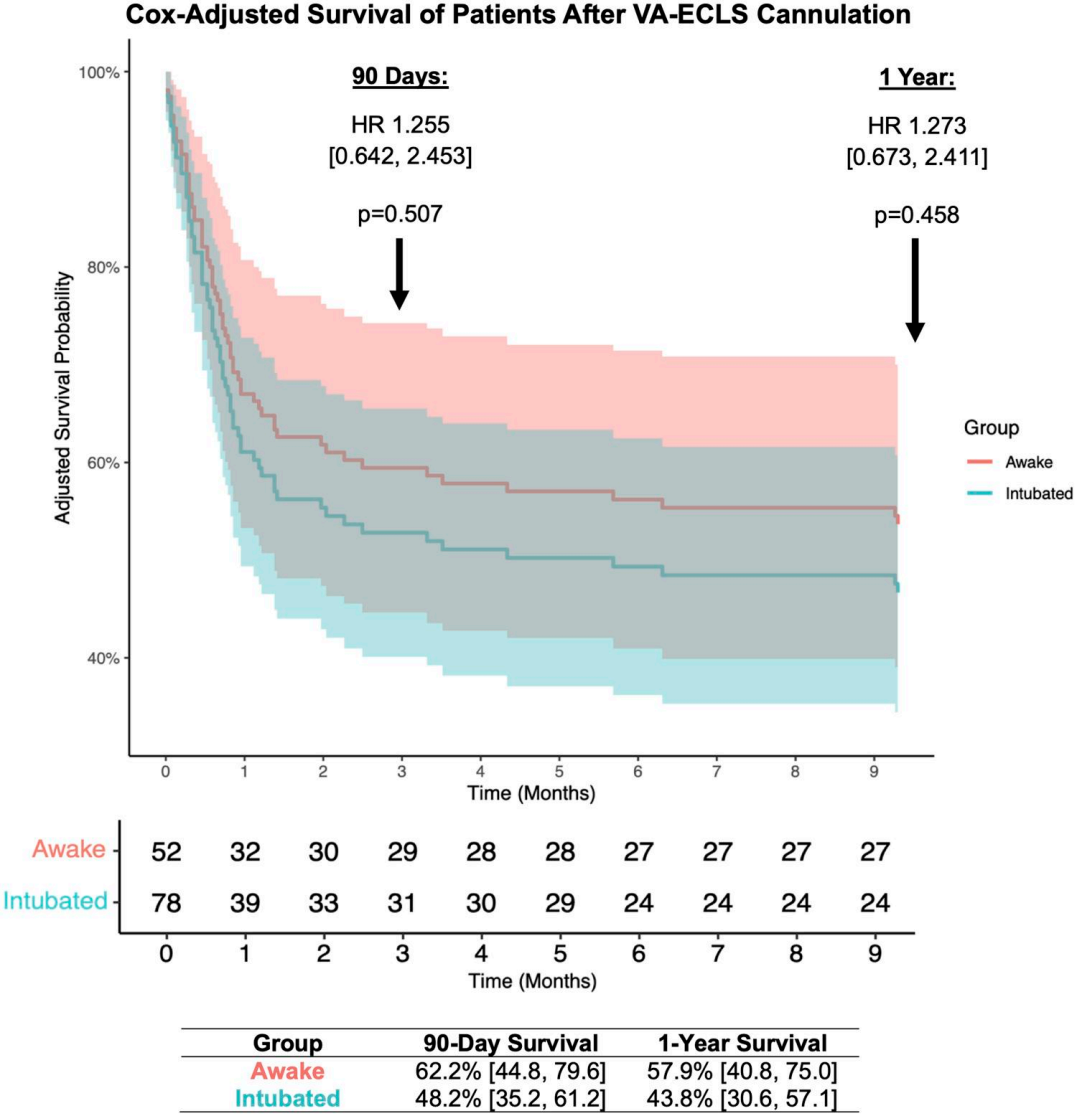
Adjusted survival curves for Cox proportional hazards model of 90-day and 1-year all-cause mortality after VA-ECLS cannulation among inverse probability treatment weighted awake and intubated patient cohorts. Corresponding HR [95% CI] and *P*-values are displayed, along with table showing percentage surviving with 95% CI at each time point. CI: confidence interval; HR: hazard ratio; VA-ECLS: veno-arterial extracorporeal life support.

Multivariable Cox regression analysis of adjusted cohorts found intubation status at time of cannulation to not be a significant risk factor for 90-day (hazard ratio [95% CI]: 1.255 [0.642, 2.453], *P* = 0.507) or 1-year mortality (hazard ratio [95% CI]: 1.273 [0.673–2.411], *P* = 0.458) when adjusting for SCAI class, diabetes, coronary artery disease, smoking and lactate (Table 3). Overall survival at 90 days (62.2% vs 48.2%, *P* = 0.507) and 1 year (57.9% vs 43.8%, *P* = 0.458) post-cannulation was comparable between awake and intubated patients in Cox-adjusted analysis (Fig. 1). Moreover, intubation status at time of cannulation was found to be associated with the need for tracheostomy (odds ratio [95% CI]: 2.963 [1.033, 8.495], *P* = 0.045) but not for developing pneumonia (odds ratio [95% CI]: 0.982 [0.423, 2.280], *P* = 0.967) during or after VA-ECLS course in a multivariate logistic regression model adjusted for age, smoking and chronic obstructive pulmonary disease history (Supplemental Table S3).

**Table 3:** Multivariable Cox regression analysis of characteristics on 90-day and 1-year all-cause mortality after VA-ECLS in inverse probability treatment weighted cohorts

Time point	Variable	HR (95% CI)	*P*-value
90 days	Intubated at time of cannulation	1.255 (0.642–2.453)	0.507
	Diabetes	1.023 (0.558–1.880)	0.939
	Coronary artery disease	1.044 (0.556–1.962)	0.893
	Smoking	1.209 (0.633–2.311)	0.565
	Lactate (pre-cannulation)	0.922 (0.844–1.009)	0.079
	SCAI stage		
	C	Reference	N/A
	D	0.949 (0.451–1.997)	0.891
	E	4.124 (1.896–8.972)	<0.001*
1 year	Intubated at time of cannulation	1.273 (0.673–2.411)	0.458
	Diabetes	1.054 (0.591–1.881)	0.858
	Coronary artery disease	1.059 (0.575–1.950)	0.855
	Smoking	1.233 (0.665–2.287)	0.507
	Lactate (pre-cannulation)	0.926 (0.848–1.011)	0.084
	SCAI stage		
	C	Reference	N/A
	D	1.009 (0.492-2.070)	0.981
	E	4.065 (1.886-8.764)	<0.001*

CI: confidence interval; HR: hazard ratio; SCAI: Society for Cardiovascular Angiography and Interventions classification of cardiogenic shock.

* *P* < 0.05.

**Figure 2:**
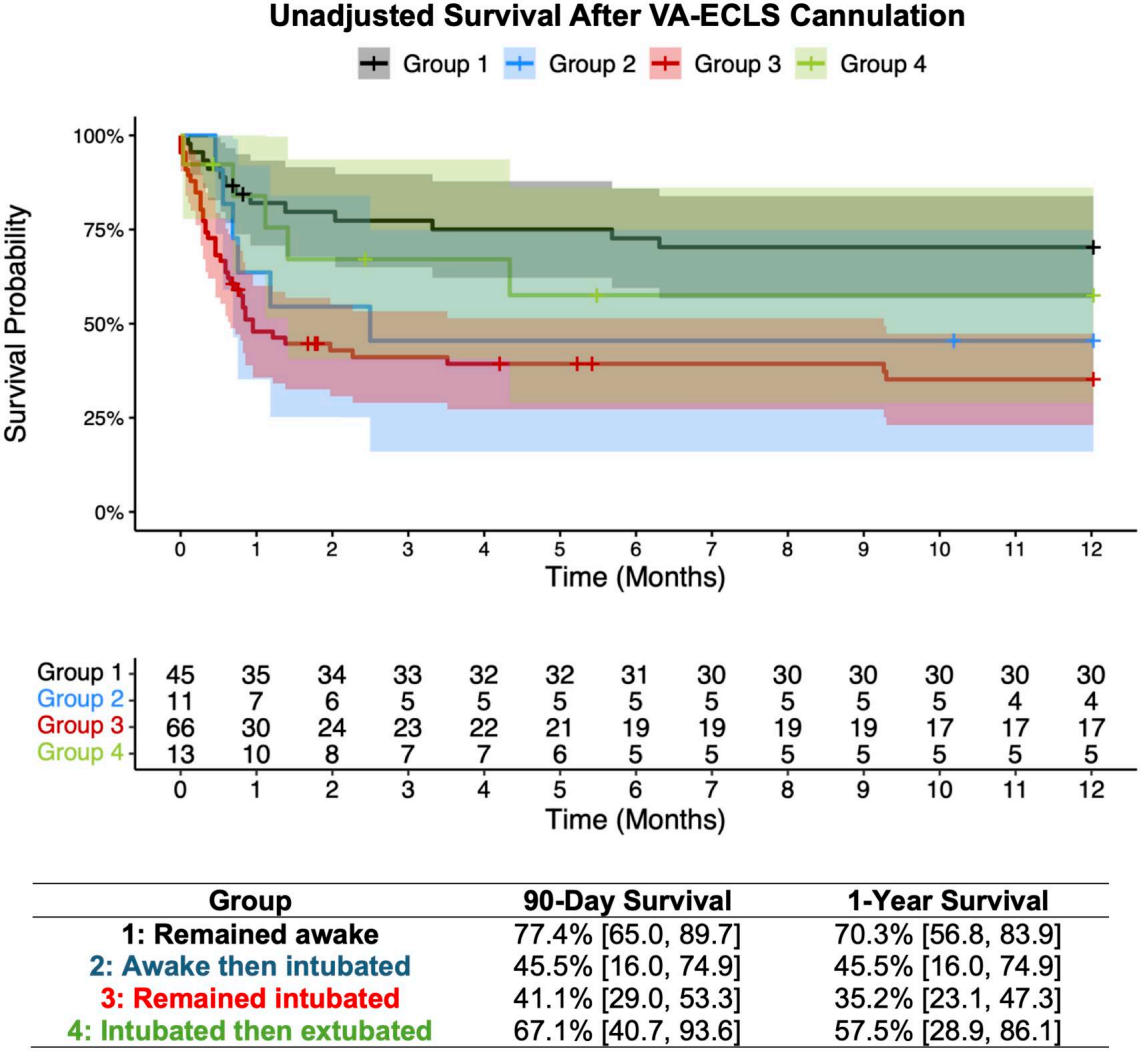
Unadjusted overall survival at 90 days and 1 year after VA-ECLS cannulation in patients stratified into four subgroups: remained awake during entire VA-ECLS course (‘group 1’), awake during cannulation then emergently intubated before decannulation (‘group 2’), remained intubated during entire VA-ECLS course (‘group 3’), and intubated during cannulation then extubated before decannulation (‘group 4’). The table of percentage surviving (95% CI) at each time point is displayed. CI: confidence interval; VA-ECLS: veno-arterial extracorporeal life support

As death is a competing risk for cross-over of intubation status in each cohort, information on the following four subgroups is provided for descriptive purposes only: (i) 45 patients remained awake during entire VA-ECLS course, (ii) 11 patients were awake during cannulation then underwent emergent intubation before decannulation, (iii) 66 patients remained intubated during entire VA-ECLS course and (iv) 13 patients were intubated during cannulation then extubated before decannulation. Table 4 displays the full baseline characteristics of the four subgroups. Within the awake crossover subgroup (*n* = 11), reasons for requiring emergent intubation are listed in Supplementary Material, Table S4. Patients who remained awake had numerically higher rates of myocardial recovery (26.7% vs 9.1%) and lower rates of in-hospital mortality (26.7% vs 54.5%) compared to awake patients who eventually required emergent intubation. On the other hand, patients who remained intubated had numerically decreased rates of myocardial recovery (36.4% vs 53.8%) and increased rates of in-hospital mortality (60.6% vs 30.8%), acute kidney injury (42.4% vs 0.0%) and continuous veno-venous hemofiltration initiation (39.4% vs 16.7%) during their VA-ECLS run compared to patients who were initially intubated then extubated before decannulation (Table 5). Patients who remained awake during their entire VA-ECLS duration had the highest rates of 90-day and 1-year survival (77.4%, 70.3%), while those who were awake then intubated (45.5%, 45.5%) and those who remained intubated (41.1%, 35.2%) experienced the worst survival (Fig. 2).

**Table 4:** Baseline characteristics of unadjusted VA-ECLS patients stratified into 4 groups: (i) remained awake during the entire VA-ECLS course, (ii) awake during cannulation then emergently intubated before decannulation, (iii) remained intubated during the entire VA-ECLS course and (iv) intubated during cannulation then extubated before decannulation

Variable	Remained awake (*n* = 45)	Awake then intubated (*n* = 11)	Remained intubated (*n* = 66)	Intubated then extubated (*n* = 13)
Age	53.0 [43.0, 63.0]	56.0 [53.5, 66.0]	62.0 [52.5, 68.0]	59.0 [55.0, 61.0]
Male gender	29 (64.4)	8 (72.7)	46 (69.7)	8 (61.5)
BMI	25.9 [22.3, 30.4]	28.0 [22.9, 28.5]	31.0 [27.1, 35.7]	30.0 [23.5, 31.7]
BSA	1.9 [1.8, 2.2]	1.8 [1.8, 2.0]	2.0 [2.0, 2.2]	2.0 [1.8, 2.2]
Coronary artery disease	21 (46.7)	3 (27.3)	40 (60.6)	6 (46.2)
Congestive heart failure	30 (66.7)	8 (72.7)	32 (48.5)	7 (53.8)
Hyperlipidaemia	25 (55.6)	6 (54.5)	44 (66.7)	10 (76.9)
Diabetes	17 (37.8)	4 (36.4)	25 (37.9)	6 (46.2)
Hypertension	36 (80.0)	7 (63.6)	51 (77.3)	10 (76.9)
COPD	2 (4.4)	1 (9.1)	6 (9.1)	1 (7.7)
Chronic kidney disease	19 (42.2)	6 (54.5)	21 (31.8)	6 (46.2)
Smoking	22 (48.9)	4 (36.4)	29 (43.9)	8 (61.5)
Prior CVA	8 (17.8)	1 (9.1)	9 (13.6)	1 (7.7)
Aetiology of shock				
Acute MI	3 (6.7)	0 (0.0)	25 (37.9)	1 (7.7)
ADHF	30 (66.7)	6 (54.5)	19 (28.8)	7 (53.8)
Pulmonary	6 (13.3)	1 (9.1)	8 (12.1)	1 (7.7)
Ventricular arrhythmia	1 (2.2)	0 (0.0)	7 (10.6)	1 (7.7)
Myocarditis	3 (6.7)	2 (18.2)	1 (1.5)	1 (7.7)
Other	2 (4.4)	2 (18.2)	6 (9.1)	2 (15.4)
IABP	26 (57.8)	7 (63.6)	19 (28.8)	4 (30.8)
Impella	8 (17.8)	2 (18.2)	26 (39.4)	1 (7.7)
SCAI stage				
C	6 (13.3)	3 (27.3)	16 (24.2)	4 (30.8)
D	37 (82.2)	6 (54.5)	25 (37.9)	4 (30.8)
E	2 (4.4)	2 (18.2)	25 (37.9)	5 (38.5)
Echocardiography				
Moderate/severe MR	11 (50.0)	4 (100.0)	11 (35.5)	3 (42.9)
LVEF (%)	15.0 [15.0, 29.0]	10.0 [10.0, 15.0]	20.0 [15.0, 30.0]	25.0 [17.0, 60.0]
LVEDD (cm)	6.0 [4.6, 7.1]	8.1 [7.4, 8.8]	5.7 [5.0, 6.7]	4.8 [4.0, 5.6]
Haemodynamics				
MAP (mmHg)	72.0 [65.0, 78.5]	70.5 [64.0, 74.8]	71.0 [66.0, 80.0]	67.5 [64.2, 86.8]
CO (Fick)	3.0 [2.5, 3.9]	1.7 [1.7, 1.7]	3.8 [3.3, 4.6]	4.5 [3.6, 5.1]
CI (Fick)	1.6 [1.2, 2.0]	1.0 [1.0, 1.1]	1.9 [1.5, 2.3]	1.8 [1.6, 2.3]
CVP (mmHg)	17.0 [14.0, 21.0]	21.0 [15.0, 26.0]	17.5 [14.0, 23.0]	16.0 [13.0, 20.0]
MPAP (mmHg)	37.0 [28.0, 50.0]	41.0 [40.0, 46.5]	33.0 [25.0, 41.2]	26.0 [23.8, 34.2]
PCWP (mmHg)	27.0 [24.5, 35.0]	27.0 [25.5, 32.0]	27.5 [24.0, 32.8]	22.5 [15.8, 29.2]
PVR (Woods units)	1.6 [1.3, 1.8]	NA	1.7 [1.1, 2.2]	2.8 [2.8, 2.8]
Labs				
P_a_CO_2_ (mmHg)	33.3 [29.5, 38.8]	36.9 [28.3, 40.6]	38.0 [33.0, 42.3]	36.2 [34.3, 40.0]
SvO_2_ (%)	45.6 [39.7, 57.1]	44.4 [31.0, 57.9]	59.0 [49.9, 69.4]	48.2 [44.1, 64.6]
P_a_O_2_/FiO_2_	295.2 [192.5, 381.0]	376.2 [170.0, 390.5]	168.7 [103.0, 318.5]	316.6 [240.8, 387.9]
Lactate	2.0 [1.3, 3.8]	2.2 [1.1, 7.6]	3.2 [1.7, 6.6]	1.9 [1.4, 7.1]
Hematocrit	34.4 [31.2, 38.9]	38.4 [29.5, 42.1]	32.7 [27.3, 39.9]	29.8 [26.3, 39.2]
eGFR	47.8 [34.3, 68.3]	40.0 [24.5, 53.2]	38.5 [27.6, 57.2]	47.9 [37.0, 100.9]

Categorical data are reported as value (%). Continuous data are reported as median [interquartile range].

ADHF: acute decompensated heart failure; BMI: body mass index; BSA: body surface area; CI: cardiac index; CO: cardiac output; CVA: cerebrovascular accident; CVP: central venous pressure; COPD: chronic obstructive pulmonary disease; eGFR: estimated glomerular filtration rate; ECLS: extracorporeal life support; IABP: intra-aortic balloon pump; LVEDD: left ventricular end-diastolic diameter; LVEF: left ventricular ejection fraction; MAP: mean arterial pressure; MI: myocardial infarction; MPAP: mean pulmonary arterial pressure; P_a_CO_2_: partial pressure of arterial carbon dioxide; PCWP: pulmonary capillary wedge pressure; PVR: pulmonary vascular resistance; SvO_2_: mixed venous oxygen saturation; P_a_O_2_/FiO_2_: partial pressure of arterial oxygen to fraction of inspired oxygen ratio; SCAI: Society for Cardiovascular Angiography and Interventions classification of cardiogenic shock; SMD: standardized mean difference.

**Table 5:** Clinical outcomes of unadjusted VA-ECLS patients stratified into four groups: (i) remained awake during the entire VA-ECLS course, (ii) awake during cannulation then emergently intubated before decannulation, (iii) remained intubated during the entire VA-ECLS course and (iv) intubated during cannulation then extubated before decannulation

Outcome	Remained awake (*n* = 45)	Awake then intubated (*n* = 11)	Remained intubated (*n* = 66)	Intubated then extubated (*n* = 13)
VA-ECLS duration (days)	6.0 [4.0, 10.0]	9.0 [6.5, 19.0]	7.0 [4.0, 9.0]	10.0 [6.0, 12.0]
% of ECLS course spent intubated	NA	66.7 [26.1, 80.2]	NA	75.0 [42.9, 90.9]
Destination at discharge				
Death	12 (26.7)	6 (54.5)	40 (60.6)	4 (30.8)
Recovery	12 (26.7)	1 (9.1)	24 (36.4)	7 (53.8)
Transplant	12 (26.7)	2 (18.2)	1 (1.5)	1 (7.7)
VAD	9 (20.0)	2 (18.2)	1 (1.5)	1 (7.7)
In-hospital mortality	12 (26.7)	6 (54.5)	40 (60.6)	4 (30.8)
Required LV unloading	26 (57.8)	9 (81.8)	40 (60.6)	5 (38.5)
Adverse events				
Distal perfusion cannula	16 (35.6)	4 (36.4)	35 (53.0)	8 (61.5)
Limb ischaemia surgery	0 (0.0)	0 (0.0)	1 (1.5)	0 (0.0)
CVVH initiation	9 (20.0)	1 (9.1)	26 (39.4)	2 (16.7)
CVA	6 (13.3)	1 (9.1)	4 (6.1)	2 (15.4)
AKI	12 (26.7)	3 (27.3)	28 (42.4)	0 (0.0)
Bleeding	15 (33.3)	8 (72.7)	28 (42.4)	5 (38.5)
PNA during ECLS	6 (13.3)	2 (18.2)	16 (24.2)	3 (23.1)
PNA during or after ECLS	20 (44.4)	4 (36.4)	30 (45.5)	4 (30.8)
Trach during ECLS	2 (4.4)	0 (0.0)	2 (3.0)	1 (7.7)
Trach during or after ECLS	7 (15.9)	2 (18.2)	22 (33.8)	1 (7.7)

Categorical data are reported as value (%). Continuous data are reported as median [interquartile range].

AKI: acute kidney injury; CVA: cerebrovascular accident; CVVH: continuous veno-venous hemofiltration; ECLS: extracorporeal life support; PNA: pneumonia; VAD: ventricular assist device; SMD: standardized mean difference.

## DISCUSSION

We present our experience with VA-ECLS for non-intubated adult patients with RCS at a high-volume ECLS center. Our results not only demonstrate that awake VA-ECLS is feasible in carefully selected patients with RCS, but also suggest safety of this approach given similar short-term survival and rates of VA-ECLS complications, such as limb ischaemia, renal dysfunction, stroke and bleeding, compared to their intubated counterparts.

Literature on awake VA-ECLS for RCS previously consisted of only case reports, paediatric populations and cohorts with very small sample sizes until recently, when Montero *et al.* [18] published their experience with awake VA-ECLS. Here, we report our institution’s experience as one of only two studies with a substantial cohort size in this topic. Similar to their study, we adjusted for several pre-cannulation differences between awake and intubated cohorts given that intubated patients likely had more severe underlying illness. Their propensity score matching techniques resulted in a final sample size of 51 subjects per cohort, while we report a larger cohort. Interestingly, our experience contrasted with theirs in that they observed significantly lower rates of mortality and in-hospital adverse events in their awake cohort while we observed no significant differences in ours. However, the definition of ‘awake ECLS’ varies widely across existing literature, as there is no consensus on how long a patient should be non-intubated in order to be considered ‘awake’ during their ECLS course. Montero *et al.* defined ‘awake’ as spending less than 50% of their VA-ECLS run on mechanical ventilation, while our study focused on the feasibility and outcomes of awake status at the time of VA-ECLS insertion. Given that 11 patients in our awake cohort ‘crossed over’ to require emergent intubation at some point during their VA-ECLS run, we also included sub-analyses where cross-overs were examined separately. We found that awake patients who were later intubated experienced numerically higher rates of in-hospital mortality, as expected given that a new intubation requirement would often follow clinical decompensation.

Emerging literature suggests advantages of not only awake ECLS cannulation, but also of avoiding of any subsequent intubation after cannulation (‘keep them awake’) [13, 14]. Additionally, the Extracorporeal Life Support Organization 2021 guidelines state that ‘extubation on ECMO should be considered’ due to risks of mechanical ventilation and sedation [19]. Sedation often leads to increased vasopressor requirements, resulting in vasoconstriction that can increase myocardial oxygen demand and risk for peripheral ischaemia and necrosis. Mechanical ventilation itself can also lead to haemodynamic deterioration in right-sided heart failure by decreasing right ventricular preload and increasing afterload [20]. Moreover, avoidance of intubation allows for normal feeding without nasogastric tube, increased mobilization, real-time neurological monitoring and avoidance of muscle wasting [7]. Some retrospective studies have suggested the safety and feasibility of early mobilization on ECLS, including ambulation, even for patients with femoral cannulation [21, 22]. The advantages of avoiding intubation may not only be beneficial during VA-ECLS itself, but could also positively impact their outcomes after advanced heart failure therapies such as VAD or transplantation. Finally, aside from clinical benefits, remaining extubated can also allow patients to participate in medical decision-making and discussions about goals of care during their critical illness. Therefore, patients without indications for mechanical ventilation or sedation should be considered for this approach. The SCAI classification of cardiogenic shock may potentially serve as a guiding tool in assessing whether awake VA-ECLS could be feasible for a patient, although more longitudinal studies are required to elucidate specific patient characteristics that are most appropriate for awake cannulation.

This study has several limitations to highlight. As a retrospective study, it is not possible to fully control for baseline differences in patient comorbidities and illness severity. We attempted to mitigate this limitation by adjusting for differences in the variables available to us through IPTW and subsequent multivariable regression modelling techniques. However, after these efforts, some differences still remained between the groups as modelling techniques are inevitably limited. There may be confounding from unmeasured variables such as severity and chronicity of certain co-morbidities, nutritional status, psycho-social factors and genetic differences. Although we present one of the largest studies of awake VA-ECLS from a high-volume ECLS center, statistical power still remains limited so many of the differences not found to be statistically significant could become significant with a larger sample size. The results presented in this study should be interpreted in the context of limited information available.

In summary, our institutional experience reported in this study suggests that awake VA-ECLS can be safe, feasible and effective in select patients with RCS. Similar studies should be repeated in the future using larger cohorts as experience with the awake VA-ECLS approach grows over time.

## Supplementary Material

ivae148_Supplementary_Data

## Data Availability

The data underlying this article will be shared on reasonable request to the corresponding author.

## ABBREVIATIONS

CI: Confidence interval
COPD: Chronic obstructive pulmonary disease
IPTW: Inverse probability of treatment weighting
RCS: Refractory cardiogenic shock
SCAI: Society for Cardiovascular Angiography and Interventions
SMD: Standardized mean difference
VA-ECLS: Veno-arterial extracorporeal life support
VAD: Ventricular assist device
