# Supplementary material for: Feasibility of veno-arterial extracorporeal life support in awake patients with cardiogenic shock
Source: Interdiscip Cardiovasc Thorac Surg. 2024 Aug 20;39(2):ivae148. doi: 10.1093/icvts/ivae148 (PMC11344587; doi:10.1093/icvts/ivae148)
Supplement: ivae148_Supplementary_Data [file ivae148_supplementary_data.docx]

**Supplemental Table S1.** Missing baseline values excluded from analysis.

| **Variable** | **Percent Missing** |
| --- | --- |
| P_a_CO_2_ | 25.9% |
| SvO_2_ | 40.7% |
| P_a_O_2_/FiO_2_ ratio | 36.3% |
| Cardiac output (Fick) | 72.6% |
| Cardiac index (Fick) | 67.4% |
| Central venous pressure | 31.9% |
| Mean systemic arterial pressure | 17.0% |
| Mean pulmonary arterial pressure | 34.8% |
| Pulmonary vascular resistance | 88.1% |
| Pulmonary capillary wedge pressure | 68.9% |
| Mitral regurgitation | 52.6% |
| Left ventricular ejection fraction | 48.1% |
| Left ventricular end-diastolic diameter | 60.7% |

**Supplemental Table S2.** R statistical packages used in this study.

| **Analysis** | **R Package(s)** |
| --- | --- |
| Baseline characteristics | “tableone” |
| Inverse probability of treatment weighting | “tidyverse”, “MatchIt”, “survey” |
| Propensity score density plot | “ggplot2”, “hrbrthemes”, “dplyr”, “tidyr”, “viridis” |
| SMD distribution plot | “reshape2” |
| Kaplan-Meier analysis | “survival”, “survminer” |
| Multivariable analyses | “adjustedCurves”, “riskRegression”, “TH.data”, “lubridate”, “ggsurvfit”, “gtsummary”, “tidycmprsk”, “condSURV”, “pammtools”, “scales”, “aod” |

**Supplemental Table S3.** Univariate and multivariate logistic regression analysis of risk factors for developing pneumonia or requiring tracheostomy during or after VA-ECLS in inverse probability treatment weighted cohorts.

| **Outcome** | **Selected Variables** | **Univariate analysis**  **OR (95% CI)** | **p-value** | **Multivariate analysis**  **OR (95% CI)** | **p-value** |
| --- | --- | --- | --- | --- | --- |
| Pneumonia | Intubated at time of cannulation | 0.943 (0.405-2.198) | 0.893 | 0.982 (0.423-2.280) | 0.967 |
|  | Age | 0.987 (0.960-1.015) | 0.374 | 0.991 (0.963-1.020) | 0.525 |
|  | Smoking | 0.970 (0.429-2.190) | 0.941 | 1.032 (0.462-2.306) | 0.939 |
|  | COPD | 0.432 (0.095-1.956) | 0.278 | 0.494 (0.105-2.326) | 0.374 |
| Tracheostomy | Intubated at time of cannulation | 2.565 (0.974-6.758) | 0.059 | 2.963 (1.033-8.495) | **0.045*** |
|  | Age | 0.992 (0.958-1.026) | 0.626 | 0.986 (0.950-1.023) | 0.456 |
|  | Smoking | 1.914 (0.767-4.776) | 0.167 | 2.343 (0.864-6.351) | 0.097 |
|  | COPD | 0.971 (0.171-5.522) | 0.973 | 0.803 (0.116-5.552) | 0.825 |

COPD, chronic obstructive pulmonary disease; CI, confidence interval; OR, odds ratio. *p < 0.05

**Supplemental Table S4.** Reasons for emergent intubation, timing relative to ECLS cannulation date, and duration spent intubated in awake crossover subgroup (n=11).

| **Patient** | **Intubation Reason** | **Days After ECLS Initiated** | **Days Intubated** |
| --- | --- | --- | --- |
| A | Worsening mental status of unknown etiology | 0 | 13 |
| B | Suspected aspiration | 0 | 8 |
| C | Respiratory fatigue in the setting of acidosis | 0 | 2 |
| D | Worsening pulmonary edema | 0 | 2 |
| E | Aspiration pneumonitis | 1 | 8 |
| F | Persistent ventricular tachycardia | 1 | 2 |
| G | Worsening mental status of unknown etiology | 1 | 4 |
| H | Acute respiratory distress syndrome | 6 | 15 |
| I | Suspected pneumonia | 11 | 3 |
| J | Stroke | 13 | 4 |
| K | Suspected seizure | 20 | 3 |

**Supplemental Figure S1.** Distribution of propensity scores in each cohort before inverse probability of treatment weighting.

**Supplemental Figure S2.** Distribution of standardized mean difference (SMD) values before and after inverse probability of treatment weighting.
